# Role of *Aeromonas hydrophila* Flagella Glycosylation in Adhesion to Hep-2 Cells, Biofilm Formation and Immune Stimulation

**DOI:** 10.3390/ijms151221935

**Published:** 2014-11-28

**Authors:** Susana Merino, Markus Wilhelms, Juan M. Tomás

**Affiliations:** Departamento de Microbiología, Facultad de Biología, Universidad de Barcelona, Diagonal 643, 08071 Barcelona, Spain; E-Mails: smerino@ub.edu (S.M.); mwilhelms@ub.edu (M.W.)

**Keywords:** *O*-flagellin glycosylation, adhesion, biofilm, immune stimulation

## Abstract

**Abstract:** Polar flagellin proteins from *Aeromonas hydrophila* strain AH-3 (serotype O34) were found to be *O*-glycosylated with a heterogeneous heptasaccharide glycan. Two mutants with altered (light and strong) polar flagella glycosylation still able to produce flagella were previously obtained, as well as mutants lacking the O34-antigen lipopolysaccharide (LPS) but with unaltered polar flagella glycosylation. We compared these mutants, altogether with the wild type strain, in different studies to conclude that polar flagella glycosylation is extremely important for *A. hydrophila* adhesion to Hep-2 cells and biofilm formation. Furthermore, the polar flagella glycosylation is an important factor for the immune stimulation of IL-8 production via toll receptor 5 (TLR5).

## 1. Introduction

The mesophilic *Aeromonas* *hydrophila* motility is based on a single polar flagellum expressed constitutively. The flagella morphogenesis in *A. hydrophila*, as in other Gram-negative bacteria, is a complex phenomenon that requires coordinated expression of more than 50 genes encoding structural subunits, regulatory proteins and chemo-sensor machinery. The motility by flagella represents an important advantage in moving towards favorable conditions or in avoiding detrimental environments [1], and it allows in this case mesophilic *Aeromonas* to compete with other microorganisms. These bacteria are ubiquitous water-borne bacteria, considered opportunistic pathogens of both aquatic and terrestrial animals, some species being associated with gastrointestinal and extraintestinal human diseases [2].

Some of the *A. hydrophila* strains (about 60%) are able to induce new flagella (named lateral) when grown in solid or semisolid surfaces [3]. The morphogenesis of the *A. hydrophila* lateral flagella also involves more than 35 different genes encoding structural subunits, regulatory proteins and chemo-sensor machinery [4]. Both, the polar and the lateral flagella in these strains are involved in adherence to biotic or abiotic surfaces and to biofilm formation as has been previous documented [5]. Glycosylation, either *N* or *O* linked, is increasingly being observed in bacteria (reviewed in [6,7,8]), with the most commonly reported bacterial glycoproteins being flagellins and pili. Frequently, the linkage of the glycan to the proteins is an *O*-linkage of the sugar moiety to the hydroxyl oxygen of serine (Ser) or threonine (Thr) residues [6]. We recently demonstrated that polar and lateral flagellin proteins from *A. hydrophila* strain AH-3 (serotype O34) are glycosylated with different carbohydrate moieties [9]. The lateral flagellin is modified at three sites in *O*-linkage, with a single monosaccharide shown to be a pseudaminic acid derivative of 376 Da. The polar flagellin is *O*-glycosylated by a heptasaccharide linked through a 376 Da pesudaminic acid type sugar. This glycan chain is composed by the mentioned 376 Da sugar, plus the hexasaccharide (2-Hexoses, 3-*N*-acetylhexosamines unsubstituted or substituted with phosphate and methylgroups, and an unknown moiety 102 Da) [9]. The 376 Da sugar is a derivative of pseudaminic acid, and the mutants obtained in the pseudaminic acid biosynthetic pathway abolish the production of *A. hydrophila* polar and lateral flagella [9]. The heptasaccharide polar flagellin glycan is different from the *A. hydrophila* O34 antigen lipopolysaccharide (LPS) [10]. Additionally, this polar flagellin glycan is biosynthesized through an enzyme (WecX), which links the glycan to a lipid carrier independently of the O34 antigen biosynthesis [11].

**Figure 1:**
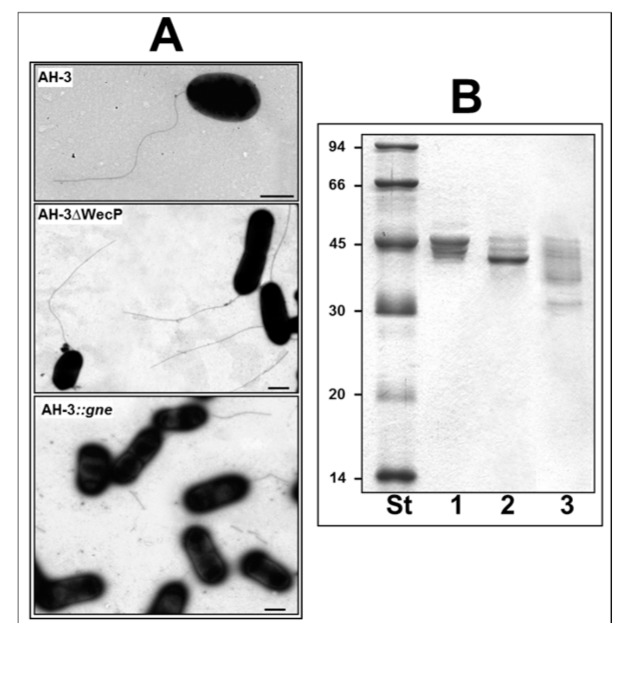
(**A**) Electron microscopy of whole cells from *A. hydrophila* strains stained according to Experimental Section. Bar represents 1 µm; (**B**) SDS-PAGE of purified polar flagellins, Coomasie blue stained. St, size standard. 1, *A. hydrophila* AH-3 (wild type); 2, *A. hydrophila* AH-3ΔWecP mutant; and 3, *A. hydrophila* AH-3:*gne* (AH-2767) mutant.

However, we previously reported two mutants with altered polar flagella glycosylation in different degrees, but still able to form flagella (Figure 1) [11]. This fact allows us in this study to compare these mutants to the wild type strain, and then to determine the role of flagellin glycosylation in several pathogenic features like bacterial adhesion or biofilm formation, as well as in immune stimulation.

## 2. Results

WecP is the enzyme that catalyzes the transfer of GalNAc-1-phosphate onto lipid carrier (undecaprenyl-phosphate) in the biosynthesis of the *A. hydrophila* AH-3 O34-antigen LPS [12]. The AH-3ΔWecP mutant, therefore, lacks the O34-antigen LPS [12]. Mutant AH-3ΔWecP is still able to produce polar flagella besides being impaired in their flagella glycosylation [11]. Mutant AH-3ΔWecP showed the same heptasaccharide chains of the wild type strain for polar flagella glycosylation but a much lower intensity. It means that several glycopeptides are not fully glycosylated in the mutant with clearly reduced amounts of phosphorylation or methylation of the sugars [11]. We consider this mutant as the one with a light reduction in polar flagella glycosylation and motility.

Gne is the enzyme able to 4-epimerize UDP-GlcNAc to UDP-GalNAc, then the mutant lacking Gne (AH-2767) should be unable to produce UDP-GalNAc [13]. Mutant AH-2767 is still able to produce polar flagella but showed a drastic reduction in glycosylation as their polar flagellin is only glycosylated by Pse or truncated glycan chains with Pse together with one or two hexoses as major moieties [11]. As we previously indicated, the HexNAc residues for polar flagella glycosylation of wild type strain AH-3 could be GalNAc. We consider this mutant as one with a strong reduction in polar flagella glycosylation and motility. This mutant lacks the O34-antigen LPS because GalNAc is a component of it [13].

**Figure 2:**
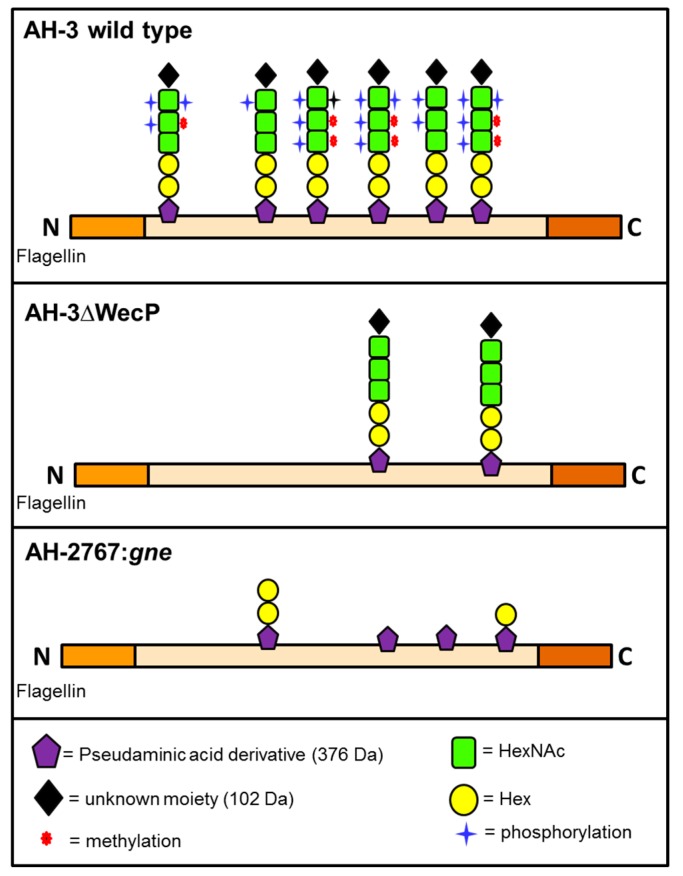
Schematic representation for the polar flagellin glycosylation of the wild type, and mutants AH-3ΔWecP and AH-2767:*gne*.

A schematic representation for the polar flagellin glycosylation of these two mutants compared to the wild type one is shown in Figure 2.

### 2.1. Adhesion to HEp-2 Cells and Biofilm Formation

In order to correlate polar flagella and their glycosylation, O34-antigen LPS, and motility with adherence to mammalian cells, we examined the interaction of several polar flagella and O34-antigen LPS mutants with cultured monolayers of HEp-2 cells. WecX is an enzyme able to link sugars to a lipid carrier responsible for the heptasaccharide glycosylation of the polar flagellin. Mutants lacking WecX are unable to produce flagella but showed no alterations in O34-antigen LPS [11]. ManC is the enzyme responsible for the isomerization of mannose-6-P to mannose-1-P guanylyl transferase. Because mannose is a component of O34-antigen LPS, the mutant (ΔManC) lacks O-antigen LPS [11,14]. Differences in adherence were calculated by determining the average number of bacteria adhering to HEp-2 cells (Table 1).

**Table 1:** Adhesion of different *A. hydrophila* serotype O34 strains to HEp-2 cells as described in Experimental section. Values presented are mean +/− SD from three independent experiments carried out in duplicates or triplicates (*n* = 6 or *n* = 9).

Strain and Characteristics	Mean No. of Bacteria/ HEp-2 Cell +/− SD	% Reduction in Adhesion ^a^
AH-3 (wild type)	18.3 +/− 2.2	-
AH-2767: *gne* mutant (O34^−^; flagella^−−^^/+^)	7.2 +/− 0.7	61 ^b^
AH-2767 + pACYC-GNE	18.0 +/− 2.5	<5
AH-3ΔWecP (WecP mutant) (O34^−^; flagella^−/+^)	9.8 +/− 1.0	46
AH-3ΔWecP + pBAD-WecP	17.9 +/− 1.7	<5
AH-3ΔManC (O34^−^ mutant)→(O34^−^; flagella^+^)	14.2 +/− 1.5	23
AH-3ΔManC + pBAD-ManC	18.7 +/− 1.9	<5
AH-3ΔPseB (flagella^−^) (O34^+^; flagella^−^)	2.2 +/− 0.6	88
AH-3ΔPseB + pBAD-PseB	17.6 +/− 2.1	<5
AH-3ΔPseBΔManC (flagella^−^, O34^−^ mutant)	<1.5	>90
AH-3:WecX (flagella^−^, O34^+^)	2.3 +/− 0.4	87
AH-3:WecX + pBAD-WecX	18.1 +/− 1.6	<5

^a^ The level of adhesion of strain AH-3 was used as 100% value; ^b^ Student’s *t* test, *p*, 0.001; ^−^, lack of the corresponding structure; ^+^, presence of the corresponding structure; −/+, light reduction of polar flagella glycosylation; ^−/−+^, strong reduction of polar flagella glycosylation.

The *A. hydrophila* wild type strain, AH-3, exhibited an adhesion value of 18.3 (18.3 ± 2.2) bacteria adhered per HEp-2 cell and a biofilm formation ability with an OD_570_ value of 1.32 (1.32 ± 0.11). Mutants lacking both flagella (polar and lateral) and O34-antigen LPS (AH-3ΔPseBΔManC) showed no adhesion to eukaryotic cells, while mutants lacking only flagella (AH-3ΔPseB or AH-3:WecX) showed a minimal adhesion value with a drastic reduction compared with the wild type. When a mutant lacking only O34-antigen LPS with unaltered flagella (AH-3ΔManC) was examined only a moderate reduction (23%) in Hep-2 cell adhesion was observed. Mutants AH-3ΔWecP and AH-2767 are devoid of O34-antigen LPS [12,13], but AH-2767 mutant showed a greater reduction in Hep-2 cell adhesion than AH-3ΔWecP, probably because their flagella glycosylation is more severely affected.

We also compared the ability of the wild type and the different mutant strains to form biofilms in microtiter plates (Table 2).

**Table 2:** Biofilm values of several *A. hydrophila* strains using the method of Pratt and Kolter as indicated in the Experimental Section. Values presented are mean +/− SD from three independent experiments carried out in triplicates (*n* = 9).

Strain and Characteristics	Value (OD_570_)
AH-3 (wild type)	1.32 +/− 0.11
AH-2767: *gne* mutant (O34^−^; flagella^−−/+^)	<0.2
AH-3ΔWecP (O34^−^; flagella^−/+^)	0.47 +/− 0.05
AH-3ΔManC (O34^−^; flagella^+^)	0.65 +/− 0.07
AH-3ΔPseB (flagella^−^)	<0.2
AH-3ΔPseBΔManC (O34^−^; flagella^−^)	<0.2
AH-3:WecX (flagella^−^)	<0.2
AH-2767: *gne* mutant + pACYC-GNE	1.30 +/− 0.15
AH-3ΔWecP + pBAD-WecP	1.29 +/− 0.13
AH-3ΔManC + pBAD-ManC	1.31 +/− 0.10
AH-3ΔPseB + pBAD-PseB	1.27 +/− 0.12
AH-3:WecX + pBAD-WecX	1.29 +/− 0.08

^−^ lack of the corresponding structure; ^+^, presence of the corresponding structure; −/+, light reduction of polar flagella glycosylation; ^−/−+^, strong reduction of polar flagella glycosylation.

The results obtained for biofilm formation show a similar overall pattern to the adhesion, when comparing the characteristics of wild type and mutant strains. However, the effects are more drastic when observing reduction in biofilm formation. Mutants lacking flagella (AH-3ΔPseB and AH-3:WecX) or flagella and O34-antigen LPS altogether (AH-3ΔpseBΔManC) are unable to form biofilms, with values <0.2, which is the limit of detection for the assay (Table 2). In the AH-3ΔManC mutant, the lack of O34-antigen LPS alone, reduced, by approximately 50%, the ability to produce biofilms (observed value of 0.65 compared with 1.32 for wild type). AH-3ΔWecP and AH-2767 mutants showed a highly reduced ability to form biofilms in comparison with the AH-3ΔManC mutant, and again the reduction is less drastic in mutant AH-3ΔWecP (value of 0.47) than in AH-2767 (>0.2). In all cases, adhesion to Hep2-cells and biofilm formation of the mutants were fully rescued by the introduction of the wild type genes (Table 1 and Table 2). No differences were observed when mutant strains harbor the plasmid vector alone.

### 2.2. IL-8 Immune Stimulation

**Figure 3:**
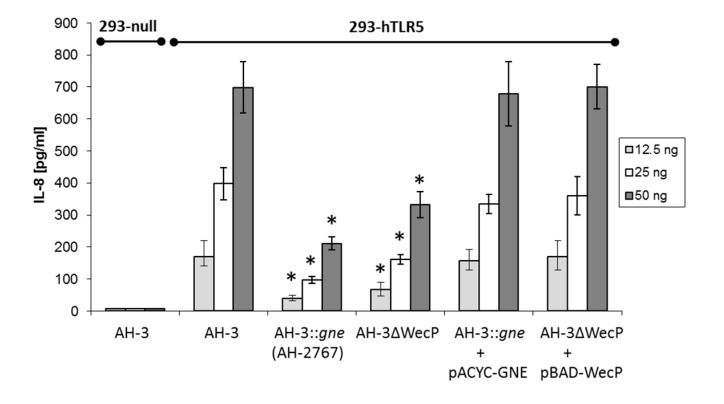
Human embryonic kidney cells (HEK293-null) and cells stably transfected with the human toll receptor 5 (TLR5)-gene (*HEK293-hTLR5*) were exposed to three different amounts of purified *A. hydrophila* AH-3 wild type or both mutants (AH-3ΔWecP and AH-2767) polar flagellins and IL-8 release was measured. The data represent the mean +/− SD of three independent experiments carried out in triplicates (*n* = 9). * *p* < 0.05, student’s *t*-test compared to the AH-3.

The HEK293 cell line was stably transfected with toll receptor 5 (TLR5) and the production of IL-8 used to assess the degree of TLR5 activation. Control HEK293-null cells stimulated with two concentrations of purified *A. hydrophila* AH-3 polar flagellins showed no IL-8 production. HEK293-hTLR5 cells stimulated with purified polar flagellins from wild type and both mutants (AH-3ΔWecP and AH-2767) showed varying levels of IL-8 production (Figure 3). Polar flagellins from AH-3ΔManC mutant lacking O34-antigen LPS are fully glycosylated, with sugars phosphorylated and methylated, showing similar values as the wild type.

The highest levels of IL-8 production were observed when cells were stimulated with wild type polar flagellin. Stimulation of cells with polar flagellin from the *gne* mutant (AH-2767) resulted in over a 70% reduction in IL-8 production relative to wild type polar flagellin. This was observed when cells were stimulated with 12.5, 25 or 50 ng of flagellin. When cells were stimulated with polar flagellin from the AH-3ΔWecP mutant, a 50% reduction in IL-8 production was observed compared to wild type one. In each case, the amount of IL-8 produced was dose dependent increasing with the flagellin concentration added. A full recovery of polar flagellin stimulated IL-8 production was observed when AH-3ΔWecP and AH-2767 mutants were rescued with the single *gne* or *wecP*, respectively (Figure 3).

No IL-8 production was obtained by HEK293-hTLR5 cells when stimulated with higher concentrations (up to 100 ng) of the nonglycosylated AH-3 polar flagellins His_6_-FlaA and His_6_-FlaB proteins obtained in *E. coli* (see Experimental Section).

## 3. Discussion

Mutants AH-3ΔWecP and AH-2767:*gne* lack the O34-antigen LPS, and our previous adhesion and biofilm studies indicate that the O34-antigen LPS is a factor for bacterial adhesion and biofilm production [13]. However, bacterial adhesions to eukaryotic cells or biofilm production are pathogenic features depending on multifactorial components [15,16].

Our current work suggests that the flagellum is a major adhesin and that motility is an important factor for adhesion. The flagella glycosylation is determinant for adhesion to eukaryotic cells or biofilm formation, as described in other mucosal pathogens [17,18,19]. The comparison between mutants only devoid of O34-antigen LPS but with complete flagella, and flagella glycosylation AH-2767 *gne* and AH-3ΔWecP mutants with altered flagella glycosylation, allows us to conclude that a full, functional polar flagellum for bacterial adhesion is only achieved when the flagella glycosylation is complete (full heptasaccharide with phosphate and methyl groups). Reduced polar flagella glycosylation is related to reduce adhesion to eukaryotic cells, being deeper in mutant with strong polar flagella glycosylation reduction (AH-2767:*gne*) than the one with light reduction (AH-3ΔWecP).

A similar, but more drastic, situation is observed when we studied the ability to produce biofilms of mutants only devoid of O34-antigen LPS but with complete flagella and flagella glycosylation and AH-2767:*gne* and AH-3ΔWecP mutants with altered flagella glycosylation. In this case the mutant with strong polar flagella glycosylation reduction (AH-2767:*gne*) is already unable to produce biofilms like mutants without flagella. The mutant with light polar flagella glycosylation reduction (AH-3ΔWecP) is still able to produce biofilm but with a clear reduction compared to the one for mutants only devoid of O34-antigen LPS.

Toll-like receptors (TLRs) are one of the key components of innate immunity. Among various types of TLRs, TLR5 is involved in recognizing bacterial flagellin and, after binding, it triggers myeloid differentiation primary response gene 88 (MyD88)-dependent signaling pathway to induce pro-inflammatory cytokines like IL-8 [20]. Our results indicate that *A. hydrophila* polar flagellin glycosylation is important for immure stimulation of IL-8 production via TLR5. Furthermore, the level of flagellin glycosylation directly correlates with the enhancement of IL-8 production. No IL-8 production in the assay described is observed when we used polar unglycosylated flagellin monomers (FlaA or FlaB) obtained in *E. coli*. It has been described that fish infected with pathogenic bacteria showed the majority of their tissues with significant enhanced expression of IL-8 for instance in the blood and intestine, and suggest the important role of TLR5 in augmenting innate immunity in fish in response to pathogenic invasion [21]. Then, it is tempting to speculate, the possible role of *A. hydrophila* AH-3 polar flagella glycosylation in cell invasion as it is a characteristic observed for the wild-type strain [22].

In conclusion, the current study starts to shed light on the biological role of flagella glycosylation not only in flagella formation and motility, either in adherence to Hep-2 cells or biofilm formation and TLR5 activation.

## 4. Experimental Section

### 4.1. Bacterial Strains, Plasmids and Growth Conditions

The bacterial strains and plasmids used in this study are listed in Table 3. *E. coli* strains were grown on Luria–Bertani (LB) Miller broth and LB Miller agar at 37 °C, while *A. hydrophila* strains were grown either in tryptic soy broth (TSB) or agar (TSA) at 30 °C. When indicated chloramphenicol (25 µg/mL), kanamycin (50 µg/mL), and tetracycline (20 µg/mL) were added to the media.

**Table 3:** Bacterial strains and plasmids used.

Strain or Plasmid	Relevant Characteristics	Reference or Source
*E. coli* strains		
DH5α	F^−^ *end A hsdR17* (rK^−^ mK^+^) *supE44 thi-1 recA1* *gyr-A96 _80lacZ*M15	[23]
BL21(λD3)	F− *ompT hsdS_B_* (r_B_^−^ m_B_^−^) *gal dcm*(λD3)	Novagen
*A. hydrophila* strains		
AH-3	O34, Wild type	[12]
AH-2767	AH-3:*gne* mutant Km^R^	[13]
AH-3ΔWecP	AH-3 WecP mutant	[12]
AH-3ΔManC	AH-3 mutant in frame unable produce O34-antigen LPS	[11]
AH-3ΔPseB	AH-3 *pseB* mutant in frame with pDM4	[9]
AH-3ΔPseFΔManC	AH-3 double mutant *pseB* and in frame unable produce O34-antigen LPS	[11]
AH-3:WecX	AH-3 *wecX* defined insertion mutant, Cm^R^	[11]
Plasmids		
pET-30 Xa/LIC	IPTG inducible expression vector Km^R^	Novagen
pET-30-FlaA	pET-30 Xa/LIC with *A. hydrophila* AH-3 *flaA*	This study
pET-30-FlaB	pET-30 Xa/LIC with *A. hydrophila* AH-3 *flaB*	This study
pBAD-ManC	pBAD33 with AH-3 *manC*	[11]
pBAD-WecP	pBAD33 with AH-3 *wecP*	[12]
pBAD-WecX	pBAD33 with AH-3 *wecX*	[11]
pACYC-GNE	pACYC184 with AH-3 *gne*	[13]

### 4.2. Motility Assays

Freshly grown bacterial colonies were transferred with a sterile toothpick into the centre of swim agar (1% tryptone, 0.5% NaCl, 0.25% agar). The plates were incubated face up for 16–24 h at 25 °C and motility was assessed by examining the migration of bacteria through the agar from the center towards the periphery of the plate. Furthermore, motility was also assessed by light microscopy observations in liquid media.

### 4.3. Transmission Electron Microscopy (TEM)

Bacterial suspensions were placed on Formvar-coated grids and negative stained with a 2% solution of uranyl acetate pH 4.1. Preparations were observed on a Hitachi 600 transmission electron microscope.

### 4.4. Flagella Purification

*A. hydrophila* AH-3 was grown in TSB for the polar flagellum purification. For the isolation of lateral flagella the strains were grown in TSA and plates washed with 100 mM Tris (pH = 7.8). Cells were in both cases collected by centrifugation at 5000× *g*, and suspended in the same Tris buffer. Flagella were removed from the cells by shearing in a vortex with a glass bar for 3–4 min, and then passing repetitively (minimum six times) through a syringe. Cells were removed by centrifugation at 8000× *g* for 30 min, and the supernatant centrifuged at 18,000× *g* for 20 min. From the remaining supernatant the flagella were pelleted by ultracentrifugation at 100,000× *g* for 60 min, and resuspended in 100 mM Tris (pH = 7.8) plus 2 mM EDTA buffer. This flagella enriched fraction was purified in a cesium chloride gradient by ultracentifugation at 60,000× *g* for 48 h. The band containing the flagella was collected, the cesium chloride removed by extensive dialysis against the same buffer (100 mM Tris, 2 mM EDTA). Purified flagella were quantified by Bradford method to evaluate protein concentration, analyzed by SDS-PAGE and Coomasie blue stained.

### 4.5. Adherence Assay to HEp-2 Cell

The adherence assay was conducted as previously described by us [24] with a slight modification of that described by Carrello *et al.* [25]. Bacteria were grown statically in brain heart infusion broth (BHIB) at 37 °C, harvested by gentle centrifugation (1600× *g*, 5 min), and resuspended in PBS (pH 7.2) at approximately 10^7^ CFU/mL *(*A_600_ = 0.07). The HEp-2 cell monolayer was infected with 1 mL of the bacterial suspension for 90 min at 37 °C in 5% CO_2_. Following infection, the non-adherent bacteria were removed from the monolayer by three washes with PBS. The remaining adherent bacteria and the monolayers were then fixed in 100% methanol for 5 min. Methanol was removed by washing them with PBS, and the HEp-2 cells with the adherent bacteria were stained for 45 min in 10% (*v*/*v*) Giemsa stain (BDH) prepared in Giemsa buffer. The coverslips were air dried, mounted, and viewed by oil immersion under a light microscope. Twenty HEp-2 cells/coverslips were randomly chosen, and the number of bacteria adhering/HEp-2 cell was recorded. The number of bacteria per infected cell was expressed as the mean +/− standard error of the mean, of a total of 75 cells for each assay. Assays were carried out in duplicates or triplicates.

### 4.6. Biofilm Formation

Quantitative biofilm formation was performed in a microtiter plate as described previously [24], by adapting the method of Pratt and Kotler [26] with minor modifications. Briefly, bacteria were grown on TSA and several colonies were gently resuspended in TSB (with or without the appropriated antibiotic); 100 µL aliquots were place in a microtiter plate (polystyrene) and incubated 48 h at 30 °C without shaking. After the bacterial cultures were poured out, the plate was washed extensively with water, fixed with 2.5% glutaraldehyde, washed once with water and stained with 0.4% crystal violet solution. After solubilization of the crystal violet with ethanol-acetone (80/20, *v*/*v*) the absorbance was determined at 570 nm.

### 4.7. Non-Glycosylated A. hydrophila Flagellins

To obtain non-glycosylated *A. hydrophila* flagellins FlaA and B we over-expressed *A. hydrophila* AH-3 *flaA* and *B* in *E. coli* using pET-30 Xa/LIC vector (Novagen, Darmstad, Germany). The *A. hydrophila* AH-3 *flaA* was amplified from AH-3 genomic DNA using primers PETflaAfor 5'-GGTATTGAGGGTGCGATGGGTCTTTAT-3ꞌ and PETflaArev 5'-AGAGGAGAGTTAGAGCCTTTGTTAGCCTTGCAGCAG-3', while the *flaB* with PETflaBfor 5'-GGTATTGAGGGTGCGATGGCCATGTACATCAACAC-3' and PETflaBrev 5'-AGAGGAGAGTTAGAGCCGTTAACCCAGCAGGGACAG-3', and the PCR products obtained ligated independently into pET-30 Xa/LIC (Novagen, Darmstadt, Germany), and electroporated into *E. coli* BL21(*λ*DE3). The His_6_-FlaA and His6-FlaB protein were overexpressed and cell lysates obtained as previously reported for other proteins [27,28]. The total membrane fraction was obtained by ultracentrifugation (200,000× *g* 30 min at 10 °C), the His_6_-FlaA and His_6_-FlaB proteins were solubilized and independently purified with a Ni^2+^-NTA agarose (Quiagen, Barcelona, Spain) as previously reported [29]. When needed the His_6_-FlaA and His_6_-FlaB proteins were concentrated using a Centriplus 10-mL YM-30 centrifugal filter device (Amicon Bioseparations, Darmstad, Germany), and typical protein preparations contained yields of 0.07-0.08 g/mL as determined by the Bio-Rad Bradford assay (Barcelona, Spain).

### 4.8. Interleukin-8 (IL-8) Assay

Human embryonic kidney cells that were stably transfected with a plasmid carrying the human TLR5-gene (*HEK293-hTLR5*) and the corresponding control cell line that only expresses endogenous levels of TLR5 (HEK293-null) were obtained from InvivoGen (San Diego, CA, USA). The cells were maintained in Dulbecco’s modified Eagle’s medium supplemented with 14.5 g/L glucose, 10% (*v*/*v*) fetal bovine serum, 50 U/mL penicillin; 50 µg/mL streptomycin, 100 µg/mL Normocin, 2 mM l-glutamine in 75 cm^3^ flasks and, without the supplementation of antibiotics, in 96-well plates for measurement of interleukin-8 (IL-8).

HEK293-hTLR5 and HEK293-null cells were seeded in 96-well plates and grown to confluence before stimulation with wild type and mutants purified flagella as indicated previously at different concentrations for 5 h. IL-8 concentration in the culture medium was determined using an enzyme-linked immunosorbent assay kit (Amersham Interleukin-8 ((h) IL-8) Human, Biotrak Elisa System; G.E. Healthcare, Buckinghamshire, UK) according to the instructions provided by the manufacturer.

### 4.9. Statistical Analysis

Results are expressed as the means standard deviations (SD) of three to four experiments. Student’s *t-*test was used to compare mean values. Differences were considered significant when *p* values were < 0.05.

## 5. Conclusions

The current study starts to shed light on the biological role of flagella glycosylation not only in flagella formation and motility, either in adherence to Hep-2 cells or biofilm formation and TLR5 activation. The importance of flagella glycosylation in flagella production or motility in terms of qualitative “on-off” has been described. This study also demonstrated the importance of complete flagella glycosylation *versus* uncompleted flagella glycosylation (quantitative) in biological activities, such as adherence to Hep-2 cells or biofilm formation. A full flagella glycosylation is necessary for maximum adherence to Hep-2 cells or biofilm formation because intermediate levels of flagella glycosylation jeopardize both biological activities. Finally, the degree of IL-8 production via TLR5 activation by purified flagellin is directly linked to the flagella glycosylation amount. A non-full flagella glycosylation reduces the IL-8 production by TLR5 activation.
